# Breastfeeding Twins: A Qualitative Study

**DOI:** 10.3329/jhpn.v31i4.20049

**Published:** 2013-12

**Authors:** Nursan Dede Cinar, Tuncay Muge Alvur, Dilek Kose, Tijen Nemut

**Affiliations:** ^1^Sakarya University School of Health Sciences, Sakarya, Turkey; ^2^Kocaeli University School of Medicine, Department of Family Medicine, Kocaeli, Turkey

**Keywords:** Breastfeeding, Mothers’ experiences, Multiple babies, Qualitative study, Turkeyitative Study

## Abstract

The purpose of this qualitative research was to explore the needs and difficulties of mothers who had multiple babies at Sakarya County by focusing on their breastfeeding experience. Ten mothers who gave birth to multiple infants participated in the study voluntarily. The framework method of data analysis was applied systematically both within and across cases, with categories and themes identified by reading transcripts of interviews. Major themes generated from focus narrative interviews are described. These themes are: willingness of mothers to breastfeed and continue, management of breastfeeding, use of pacifier, daily life, ınstructions of healthcare personnel, and advices from practice of experienced mothers. This study showed that women were aware of the importance of mother's milk for their babies. They all, somehow, made intensive efforts to breastfeed their twins. Women who expect and/or have multiple babies need much more support and guidance, which may include advice for nutritional and daily care.

## INTRODUCTION

Multiple pregnancies are often associated with prematurity and low-birthweight infants. Premature infants are prone to developing postnatal complications, including recurrent episodes of sepsis, necrotizing enterocolitis, and retinopathy of prematurity (1). Many studies showed the superiority of human milk for their infants over that of other species. Use of breastmilk is associated with decreased incidence of complications (2).

This qualitative research arose from involvement of one of the researchers (NC) in a breastfeeding education programme for mothers. During those educational sessions, the author realized that the programme was not appropriate for the women carrying multiple babies. Despite their aim of encouraging the use of human milk, medical care providers often find it difficult to provide advice and support to these families. We set out to explore the needs and difficulties of those mothers by focusing on their experiences with breastfeeding their babies.

## MATERIALS AND METHODS

### Study design and settings

This was a qualitative narrative interview-based study carried out in May 2012 at State Hospital situated in Sakarya provincial centre. Participating mothers were interviewed by first author (NC) who has field experience on breastfeeding education.

### Participants

Mothers who gave birth to multiple babies at the State Hospital and lived in the city centre were called by one member of the study group to inform about the study. Ten mothers volunteered to participate and accepted home-visits. Characteristics of the mothers and their twins are presented in the table. The age range of the mothers was 21 to 34 years, and all belonged to middle-class economic status. Four of the women were primiparae; the others had breastfeeding experience with their previous singletons. The gestational age for the multiples ranged between 33 and 39 weeks. All were born by caesarean delivery. Actual age range of the twins was 2-24 months. Hospitalization of one or more of the infants ranged from two days to four weeks after birth, with five sets discharged from hospital at the same time as their mothers.

### Ethics and data collection

Ethical Committee of the University of Sakarya approved the study. One of the researchers (DK) contacted the family by telephone and gave information about the research and made an appointment for the interview. In this study, meetings have been held together with all researchers in the study team. Data were obtained through recorded narrative interviews by NC who had conducted studies on breastfeeding and multiple babies before. Interviews were transcribed by DK and TN. Sampling continued until ongoing analysis revealed no new findings, and saturation was reached with 10 mothers.

**Table .** General information about the mothers and twins

Name of mother	Age of mother	No. of kids at home	Gestational age of twins	Actual age of twins (months)	Birthweight (g) of the first baby	Birthweight (g) of the second baby
Ayse A	34	0	40	19	3,000	2,650
Ayse B	26	1	35	24	1,900	2,100
Betül	32	1	37	6	2,500	2,250
Canan	29	1	40	18	2,500	2,600
Elmas	21	0	37	6.5	2,025	2,750
Fatma	31	1	36	14	2,420	2,200
Havva	34	2	35	6	2,200	2,000
Serap	34	1	35	16	2,590	2,650
Sevil	28	0	33	2	1,400	2,080
Zeliha	28	0	39	13	2,840	2,820

### Data analysis

The framework method of data analysis was applied systematically both within and across cases with categories and themes identified by reading the transcripts. All authors were involved in reading and analyzing the transcripts. During revision, researchers read the whole text together and judged the credibility and came to the thematic consensus. Quotations supporting the credibility were chosen for every theme.

## RESULTS

### Willingness of mothers for breastfeeding and continuity

Eight of the women declared that they were determined to breastfeed their twins. One of the mothers (Ayse A) said, “I was always crying when nursing. My nipples were cracked. I was determined to continue. Nurses told me that my milk won't be enough and advised me to give formula milk but I didn't.” Another mother (Sevil) told that she was determined to breastfeed the twins: “I didn't think about the sufficiency of my milk…I supposed that it will be enough for two or three months. On their visits, the doctor told that babies were poorly nourished and offered formula milk.” Mother Zeliha was also determined to breastfeed the twins exclusively but, after caesarean section, the nurses fed the twins formula milk due to insufficiency of the mother's milk.

The duration of exclusive breastfeeding ranged between one day and five months. The main reason for starting formula milk was the extreme concern about the babies being not completely sated. Ayse B stated, “I wish I had worked harder to breastfeed but I could not stand their crying.” Mother Zeliha said, “As I was operated, nurses gave formula milk to the babies after the delivery. After then, I gave formula milk once a day but just conscientiously… actually breastfeeding was enough.” One baby of mother Serap was hospitalized for one day due to hypoglycaemia; she stated, “I was frightened…I thought that he will be hypoglycaemic again…I gave formula milk 2-3 times a day.” Another mother (Havva) was concerned about weight of the babies. “They were small. To have the twins grow well, I gave formula milk.”

Two mothers (Canan and Elmas) exclusively breastfed their twins—one for three and the other for four months.

### Management of breastfeeding

At their hospital stays, all mothers were advised to offer one breast to one baby, the other to the second baby; the mothers were assisted for simultaneous breastfeeding. After discharge, nine mothers preferred breastfeeding individually and gave both breasts to both babies. The main reasons for this type of nursing were: (i) small size of babies, (ii) lack of assistance, (iii) share of more equitable mother's milk. Mother Fatma said, “The girl was sucking weaker than the boy, and always there was milk residue on her side. The boy was sucking the rest of milk after the girl…I nourished on their demand.” Betül stated, “I bought a twin breastfeeding pillow and tried simultaneous breastfeeding but I couldn't succeed….the babies were too small.” Ayse A said, “There was more milk on the right side…the fatty baby (3,000 g) was sucking that side; the other one was 2,650 g…I thought that was unjust…thus, I interchanged the breasts.”

### Use of pacifier

Six sets of twins did not use pacifiers but three of the mothers reported that they tried to give. The main reasons for the use of pacifier were: to soothe babies, help put babies to sleep, keep babies comforted and quiet, help them stretch the time between feeds, and as a distraction. Mother Elmas said, “I pushed to give dummy…I tried just after birth… but they didn't accept… I wanted them to be comforted and quiet…to help put babies to sleep.” Sevil stated, “It made them queasy…they don't want dummy or bottle.” Zeliha also stated, “I desired…but the girl used for two months and the boy for four months…then they quit…I hoped to be free and easy at nights at least a bit.” Canan said, “They were three months old…I started to soothe them and put them to sleep.”

### Daily life

All mothers experienced burden and difficulties in coping, especially when there was no help. Ayse B opined, “You should breastfeed them with two hours interval, the doctor told me so…their birthweigths were low (1,900 g and 2,100 g)…my elder daughter was having transfusions at that time (for thalassaemia minor)…going to hospital and all those things wore me out…milk ceased…because of distress.” Betül said, “I breastfed my elder for three years…I stressed on milk sufficiency…I thought it won't be enough for both….there was assistance but I preferred doing myself…I was exhausted…breastfeeding was taking a lot of time…while nursing one, the other was crying…I had to take the baby off the breast…I was filled with remorse.” Canan said, “I had fatigue, sleeplessness, and back pain….I am having psychological treatment…it's the fifth day…twins are bustling (18 months old). I can't go out with both…I can't spare time for myself…I feel suffocated sometimes… I can't take good care of my elder daughter (12 years old)…. She also needs psychological help…I do not want one more twin…five children are okay but one at a time.” Havva stated, “I awoke several times during the night…they were crying a lot…it was very tiring…I have continuous back pain and sleeplessness.”

### Instructions of healthcare personnel

Four of the mothers’ answer was ‘yes’ when asked about breastfeeding instructions of healthcare personnel during their prenatal visits. Extent of this instruction was not more than “give one breast to one baby; breastfeed them simultaneously.” The importance of prenatal instructions by healthcare professionals was mentioned by all mothers. Ayse A and Havva said, “Instructions should start during pregnancy…an illustrated booklet would be very good.” Sevil stated, “I always asked the nurses how to…they just answered me as I questioned…they didn't give information on their own.”

### Advice from practices of experienced mothers to the future mothers of twins

Ayse A: I recommend intake of sweet and desert, and drinking a lot of water…I advise them to be sure of themselves about breastfeeding.

Ayse B: They should give milk as long as they can…mine were ill frequently after I ceased breastfeeding…There must be a helper with them.

Betül: They should be relaxed…they should not be sleepless and stressed…there must be a support… a helper… mother's milk only is not enough.

Canan: I advise them to give mother's milk…first of all they have to be rested…I recommend breastmilk only for five or six months…a mother of twins is relieved when someone is with her….support is essential.

Elmas: If the mothers take good care of their feeding and take rest and not be stressed, their milk will be enough for three or four months…they should be patient…they would continuously breastfeed…they should not say “I have no milk.” They shouldn't worry about the sufficiency of milk…formula milk is not good.

Fatma: I suggest someone to help them with breastfeeding and housework. It is impossible to be a working mother and twin's mother…psychological help is necessary.

Havva: Help is essential.

Serap: When you have twins, milk runs out at night time…they can give mother's milk for four months…they should want to breastfeed…they should say “I will breastfeed under any circumstance”…it is very difficult at the beginning but then the mother will enjoy it.

Sevil: I advice them to adapt during pregnancy…breastfeeding is not difficult…they should set up a system.

Zeliha: Only mother's milk is enough for six months.

## DISCUSSION

### Willingness of mothers to breastfeed and continuity

Most mothers were worried fearing that their milk would not be sufficient for their twins. Also, they were not encouraged about the adequacy of their milk by healthcare professionals. Cries of babies and restlessness were interpreted as insufficient mother's milk.

In medical literature, it is mentioned that production of mother's milk is a matter of demand and supply, and milk production in mothers of twin babies would be enough for both the babies (3-5).

### Management of breastfeeding

Mothers of twins mentioned troubles due to poor sucking reflex and low weights of their babies. Rimon and Shinwell reported that breastfeeding was especially important for multiple babies who frequently have low birthweights and who are often born before term (6).

In some studies, main unfavourable conditions and issues for breastfeeding were stated as: (a) frequent association of prematurity with multiple pregnancies, (b) lack/weakness of sucking reflex, (c) neurodevelopmental failure, and (d) separation due to staying in intensive care (7,8).

Mothers were advised to offer one breast to one baby and the other to the second baby during their hospital stays but they all ended this trait in a while because of various reasons.

There is no final rule for breastfeeding multiple babies but it is mentioned that changing the breasts would help equitable nourishment (9-12). Different breasts may have different milk production and storing capacities. By alternating breasts, sufficient stimulation and equal milk production for both babies might be assured. Prenatal consultation that includes information on the importance of breastmilk clearly increases the initiation and duration of breastfeeding (13,14).

Damato *et al*. reported that supporting and educating the families with birth of multiple babies increase breastfeeding by 74% (7).

### Use of pacifier

In this study, the use of pacifier was frequent, and mothers stated different reasons for this.

The use of pacifiers and digit sucking are believed to be harmless habits and are commonly perceived as natural behaviour of infants (15). In many places in the world, especially in developing countries, the use of pacifiers in early childhood is very common (16).

It has been reported in numerous studies that the use of pacifiers shortens the breastfeeding period and, therefore, is not recommended for the breastfed babies (16-19).

Pollard *et al*. (1999) concluded that the use of pacifiers pacified the infants and, thus, caused them to fall asleep more easily (20). Kelmanson drew attention to the fact that pacifiers have been used for generations to soothe the crying infant (21).

Physicians, nurses, and midwives should suggest the parents to calm their babies primarily by gentle behaviours and attitudes. The families should be informed of the benefits of breastfeeding and the advantages and disadvantages of using pacifiers (16).

### Daily life

There was unmet need for help; no own time, full-time breastfeeding, continuous back pain, burn out, and sleeplessness as the daily-life strains among the mothers of twins were all reported in the literature (4,7,8).

In some studies, mothers stated that breastfeeding was easy and time-saving but, in others, some claimed that it was stressful and time-consuming (10,22).

For the continuity of lactation and adequate milk production, the mothers should know that mother's milk is the best nutrient for the babies and should avoid stress. Simultaneous nursing may yield time for resting (11).

In their study about the relation of breastfeeding sufficiency with maternal attachment and perceived social support, Cinar *et al*. reported high rates of breastfeeding sufficiency and maternal attachment among mothers who have intensive family support (23).

### Instructions of healthcare personnel

Great majority of the mothers stated that prenatal information about twin management, supported with illustrated booklets, would be good. The most frequent advice was “give one breast to one baby”, and this is not supported by the current medical knowledge, which is in favour of alternating breast to maintain equal stimulation and equitable mother's milk (3,9). From these data, we can say that healthcare professionals are in need of current knowledge on follow-up and counselling in multiple pregnancies.

In a study, Köse *et al*. found that nurses in neonatal inpatient clinics had insufficient knowledge about breastfeeding and nourishment of multiple babies. In the same study, 71.1% of the nurses were in the opinion that they are in need of in-service education on these issues (24).

### Advice from practices of experienced mothers of twins

Main advices from practices of experienced mothers of twins to the future mothers of twins, depending on self-experience were: (i) to rest/sleep, (ii) to get help/support, (iii) to take care of themselves/good diet and adequate liquid ingestion, and (iv) to stay away from stress. These were all mentioned as essential for nourishment/management of mothers with twin babies and are supported by the literature (3,7,8,11,25).

### Conclusions

In this qualitative study, women were aware of the importance of mother's milk for their babies. They all somehow made intensive efforts to breastfeed their twins. In spite of their efforts, they also mentioned their constant fears about the amount of milk and satiation of their babies.

The prominent troubles put into words were long duration of breastfeeding, exhaustion, having no one to help them, poor sucking, and lack of information about nourishment/nursing for the multiple babies.

Human milk is the best readily-available nutrition source for both singletons and multiples. Women who expect and/or have multiple babies need much more support and guidance, which may include advice on nutritional and daily care. Educated healthcare professionals should give relevant information about the adequacy criteria for mother's milk and encourage the mothers for breastfeeding during the early stages of the establishment of breastfeeding.

Illustrated documents about nourishment and nursing for the multiple babies should be prepared and offered to the mothers. Support of nurses and midwives to postpartum women should be available for multiple babies and their parents during the tribulations of breastfeeding.

It must be kept in mind that breastfeeding to multiple babies is a formidable and stressful challenge that needs breasting with sensitivity and sentiments. Nevertheless, partial breastfeeding is better than no mother's milk.

## ACKNOWLEDGEMENTS

Thanks to mothers who participated in this study.
